# Improving the benefit of processed EEG monitors: it’s not about the car but the driver

**DOI:** 10.1007/s10877-023-01004-6

**Published:** 2023-04-10

**Authors:** Michele Introna, Marco Gemma, Carla Carozzi

**Affiliations:** Neurointensive Care Unit, Department of Neurosurgery, Fondazione IRCCS Istituto Neurologico Carlo Besta, Milan, 20133 Italy

**Keywords:** EEG, DOA montoring, pEEG, Neuromonitoring, Perioperative care, Sedation

The article by Lichy Han and co-workers, recently published in this Journal, represents the opportunity for reasoning once again on the comparison of different processed electroencephalography (pEEG) devices and depth of anesthesia(DOA) monitors [1]. The study elegantly shows an *in silico* simultaneous comparison of three of the most used proprietary commercial monitors (PSI, SedLine® Masimo Inc, Irvine, CA; BIS, BIS VISTATM Medtronic Inc, Minneapolis, MN; Entropy, Datex Ohmeda S/5 monitor with EntropyTM Module, GE Healthcare, Chicago, IL) in a cohort of patients undergoing drug induced sleep endoscopy (DISE) with dexmedetomidine. The relative pEEG values are then compared, and ordinal logistic regression models were constructed to predict the measured Richmond Agitation-Sedation Scale (RASS) score during the procedure. All the pEEG indexes demonstrated a significant relationship with the RASS score, with increasing differences as the sedation deepened. In particular, the authors found elevated BIS values during the deepest sedation phases, possibly due to electromyography (EMG) interference, which apparently has less impact on PSI and Entropy metrics. The authors conclude that PSI and Entropy are better suited for assessing deeper levels of sedation with dexmedetomidine.

The presence of EMG on the EEG trace is generally felt as an artifact to be eliminated or reduced, because of the risk of a misleading pEEG index increase [2]. Therefore different DOA devices had been developed different filters on the acquired EEG trace (narrower than the 0.5–70 Hz of BIS) and different algorithms in order to clean the raw signal and maintain the pEEG index more stable in non-paralyzed patients [3]. The price to pay can be to lose an early warning and an ally when the EMG and pEEG value increasing can indicate pain or lightening anesthesia and risk of awakening [4] [5]. Recently Stewart et al. suggested that the BIS EMG signal could add useful information in critical care patients subjected to painful stimuli [6]. Moreover, Hight and co-workers compared five commercial DOA monitor in reading the same EEG traces of lightening anesthesia and found a pEEG concordance in only one-third of cases. Furthemore, in 31% of the cases there was at least one monitor showing an excessive hypnotic depth, whereas the EEG trace displayed an emergency-like pattern [7]. Therefore, it seems clear that the different DOA monitors can work well in some conditions or with different drugs, as demonstrated in the present work, while failing in others and that relying on the pEEG index alone constitute a risk [8]. The use itself of a dimensionless scale to express the “depth of anesthesia” has been questioned from some authors, given the risk of an oversimplification of the neurobiological background underlying consciousness. As sharply expressed by Sleigh in a famous editorial, the metaphor of the patient’s state being a “submarine” going deep should be substituted with the idea of the brain as a household switch-board [9].

It’s time to accept such a reality without falling, however, in despair.

First, the clinician should be aware of the technical differences in DOA analysis of the EEG and critically choose each monitor based on their own preferred tradeoff between sensibility and specificity (such as favoring an earlier warning of awareness, also if pEEG incorrectly suggests the presence of artifacts). Each commercial index is based in fact on unique EEG feature detection algorithms and different ways of determining noise, muscle activity, and burst suppression. The manufacturers also employed different methods for statistical validation of their indices, with most of the features patented.

**Fig. 1:**
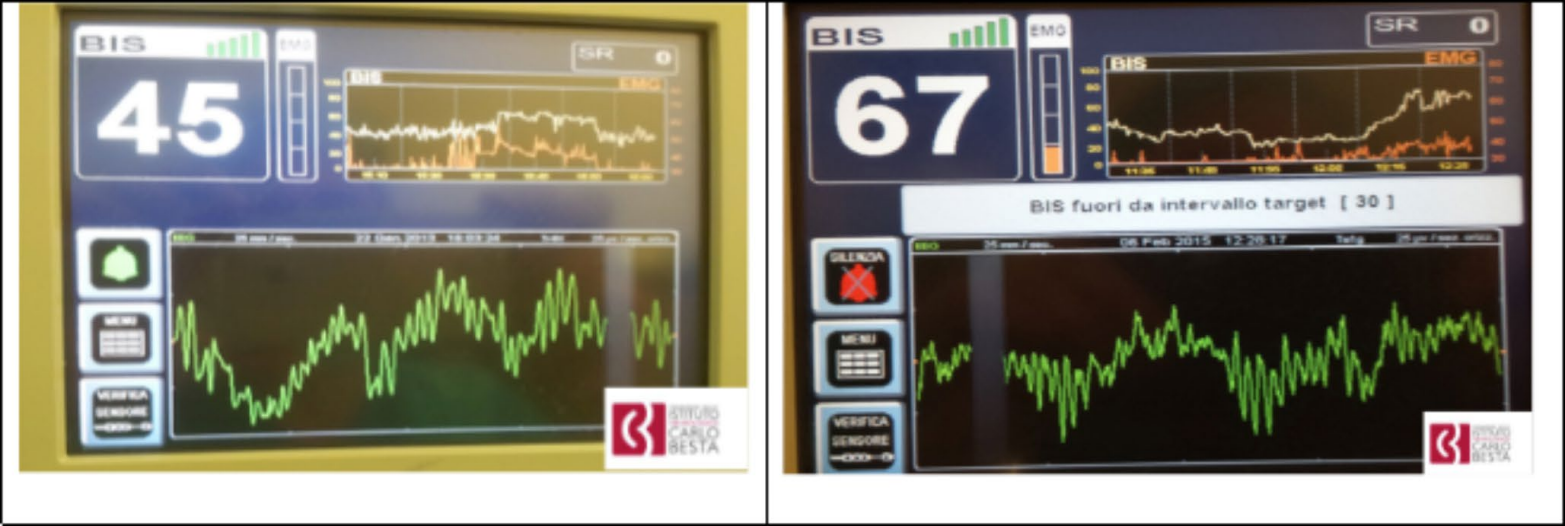
Two screenshots of the BIS monitor (BIS VISTATM Medtronic Inc, Minneapolis, MN) showing the same raw EEG pattern (delta plus alpha rhythm), respectively without (on the left) and with EMG activity (right). The patients were undergoing general anesthesia with propofol and remifentanil

Second, but not least, the clinician should learn how to interpret the raw EEG and EMG signals. Anesthesiologists are acquainted with the interpretation of electrocardiograms, which is even mandatory for a board-certified anesthesiologist. We strongly advocate that the same concept should be applied to the interpretation of raw EEG and EMG signals, in order to verify if the calculated pEEG index is coherent with the raw EEG and the clinical context (i.e. drug dosages, clinical parameters, type of patient). For example, as shown in Fig. 1, an increase of pEEG in presence of EMG activity superimposed on the typical delta/alfa EEG pattern during propofol general anesthesia should be interpreted as the result of increased noxious stimulation or artifact and not as lightening anesthesia.

The anesthesiologist has at his/her disposal many tools with enormous potential, but a specific training must be required to understand their limitations and specificities [10]. Like in the car racing world, where hyper technology cannot replace the driver’s skills in delivering the best performances, the same apply to modern medicine.

There is growing evidence that the use of pEEG also in the intensive care setting could help in avoiding under or over- sedation and possibly improve clinical outcomes, but more research is needed to validate its use in this setting [11]. The great heterogeneity introduced by the different levels of sedation and the diverse physiopathological alterations in the intensive care population makes the seek for a magic number to follow even more complex than in anesthesia [12].

The challenge would be now to step forward from the simple comparison of these monitors in different settings and eventually define the technical features and the skills needed for a safe application in a broad spectrum of clinical conditions, regardless of the specific monitor commercially available. On the other hand, a more transparent approach from the manufacturers regarding the signal filtering and manipulation would ease the process of appraisal by the anesthesiologists adopting DOA monitors in their daily practice.
